# Global Network Analysis of Neisseria gonorrhoeae Identifies Coordination between Pathways, Processes, and Regulators Expressed during Human Infection

**DOI:** 10.1128/mSystems.00729-19

**Published:** 2020-02-04

**Authors:** Ryan McClure, Ashwini Sunkavalli, Phillip M. Balzano, Paola Massari, Christine Cho, William M. Nauseef, Michael A. Apicella, Caroline A. Genco

**Affiliations:** a Biological Sciences Division, Pacific Northwest National Laboratory, Richland, Washington, USA; b Department of Immunology, Tufts University School of Medicine, Boston, Massachusetts, USA; c Department of Internal Medicine, Carver College of Medicine, The University of Iowa, Iowa City, Iowa, USA; d Department of Microbiology and Immunology, Carver College of Medicine, The University of Iowa, Iowa City, Iowa, USA; Mayo Clinic

**Keywords:** RNA-seq, *Neisseria gonorrhoeae*, network analysis, regulatory proteins, transcriptomics, global regulatory networks, human infection

## Abstract

Neisseria gonorrhoeae is a Gram-negative diplococcus that is responsible for the sexually transmitted infection gonorrhea, a high-morbidity disease in the United States and worldwide. Over the past several years, N. gonorrhoeae strains resistant to antibiotics used to treat this infection have begun to emerge across the globe. Thus, new treatment strategies are needed to combat this organism. Here, we utilized N. gonorrhoeae transcriptomic data sets, including those obtained from natural infection of the human genital tract, to infer the first global gene coexpression network of this pathogen. Interrogation of this network revealed genes central to the network that are likely critical for gonococcal growth, metabolism, and virulence, including genes encoding hypothetical proteins expressed during mucosal infection. In addition, network analysis revealed overlap in the response of N. gonorrhoeae to incubation with neutrophils and exposure to hydrogen peroxide stress *in vitro*. Network analysis also identified new targets of the gonococcal global regulatory protein Fur, while examination of the network neighborhood of genes allowed us to assign additional putative categories to several proteins. Collectively, the characterization of the first gene coexpression network for N. gonorrhoeae described here has revealed new regulatory pathways and new categories for proteins and has shown how processes important to gonococcal infection in both men and women are linked. This information fills a critical gap in our understanding of virulence strategies of this obligate human pathogen and will aid in the development of new treatment strategies for gonorrhea.

**IMPORTANCE** Neisseria gonorrhoeae is the causative agent of the sexually transmitted infection (STI) gonorrhea, a disease with high morbidity worldwide with an estimated 87 million cases annually. Current therapeutic and pharmacologic approaches to treat gonorrhea have been compromised by increased antibiotic resistance worldwide, including to the most recent FDA-approved antibiotic. New treatment strategies are urgently needed to combat this organism. In this study, we used network analysis to interrogate and define the coordination of pathways and processes in N. gonorrhoeae. An analysis of the gonococcal network was also used to assign categories to genes and to expand our understanding of regulatory strategies. Network analysis provides important insights into pathogenic mechanisms of this organism that will guide the design of new strategies for disease treatment.

## Abstract (precis)

Neisseria gonorrhoeae is the causative agent of the sexually transmitted infection (STI) gonorrhea, a disease with high morbidity worldwide with an estimated 87 million cases annually. Current therapeutic and pharmacologic approaches to treat gonorrhea have been compromised by increased antibiotic resistance worldwide, including to the most recent FDA-approved antibiotic. New treatment strategies are urgently needed to combat this organism. In this study, we used network analysis to interrogate and define the coordination of pathways and processes in N. gonorrhoeae. An analysis of the gonococcal network was also used to assign categories to genes and to expand our understanding of regulatory strategies. Network analysis provides important insights into pathogenic mechanisms of this organism that will guide the design of new strategies for disease treatment.

## INTRODUCTION

The sexually transmitted infection (STI) gonorrhea, caused by the Gram-negative pathogen Neisseria gonorrhoeae, represents the second most common reportable disease in the United States. In 2017, there were more than 550,000 reported cases of gonorrhea, and the numbers may be even higher due to underreporting. This represents an increase of 18% from the previous year and an increase of ∼75% from a historic low in 2009 (Centers for Disease Control, https://www.cdc.gov/std/stats17/gonorrhea.htm). In men, symptomatic responses to gonorrhea are characterized by a purulent discharge consisting of polymorphonuclear leukocytes (PMNs) and other immune cells (1). However, subjects infected with N. gonorrhoeae are often asymptomatic, an outcome seen more frequently in women than in men (2–5). Asymptomatic infection in women can be concerning, since these women do not seek treatment, which results in untreated gonococcal infection that can lead to several serious complications, including infertility and ectopic pregnancy (5, 6) as well as the possibility of further spreading the disease, a public health concern. While the majority of gonococcal infections are easily treated with antibiotic intervention, strains of N. gonorrhoeae resistant to several of the antibiotics commonly used to treat this infection have begun to emerge worldwide (7–11). N. gonorrhoeae remains a significant STI worldwide, and new strategies are needed to combat this infection.

Recent studies aimed at defining new therapies for gonorrhea treatment have focused on understanding pathogenic strategies used by this organism during human infection. Much of this work has focused on mimicking the environmental conditions found in the male or female genital tract and determining N. gonorrhoeae gene expression profiles in response to exposure under these conditions. Like other human pathogens, gene expression in N. gonorrhoeae is under tight control and achieved via a number of mechanisms, including classical DNA binding regulatory proteins and small regulatory RNAs (12–14). A central regulatory protein targeting several genes is the ferric uptake regulator (Fur), which controls genes involved in iron homeostasis (15–17). The gonococcal Fur protein also controls expression of the gene encoding the regulator MisR (CpxR), which confers resistance to antimicrobial peptides (18, 19), and the gene encoding the regulator OxyR, which confers resistance to oxidative stress (20, 21). Each of these regulators has a specific set of genes that it controls, although, in some cases, regulons may overlap, and it is likely that for many of these DNA binding proteins, there are additional undiscovered targets.

Several recent studies have utilized transcriptomic analysis to define the N. gonorrhoeae global response to conditions encountered during human infection. These include exposure to hydrogen peroxide (22), iron limitation (17), lack of oxygen (23), and incubation with human endothelial cells (14). Transcriptome analysis has also been performed using deletion mutants of many of the known gonococcal DNA binding regulatory proteins to better define potential regulatory pathways (17, 18, 24). Our group has also recently defined the gonococcal transcriptional response during natural mucosal infection in men and women (25, 26). Collectively, these studies have provided a comprehensive set of gene expression data, highlighting the response of the gonococcus to environmental conditions encountered during human infection.

In recent years, large data sets, such as the one described above, have been exploited to develop networks to view how genes and pathways are coordinated across conditions and to highlight metabolic and functional processes that are critical in a bacterial species, including human pathogens (27, 28). With this approach, individual transcripts or proteins represent nodes in the network, and instances of high coexpression or coabundance represent edges in the network, linking transcripts or proteins that show a high similarity in their expression profiles across a range of conditions. Network analysis studies in *Salmonella* have identified processes that are critical to pathogenesis and infection by looking for genes that occupy a central position in the network (27, 28). Those genes that have high network centrality are those that are more critical for infection. Other studies have carried out guilt-by-association (GBA) analyses to assign putative functions to uncharacterized genes based on the known genes they are connected to in the network (29, 30). Thus, network analysis can reveal critical information on functional and regulatory pathways in biological systems.

In the present study, we utilized several gonococcal transcriptomic data sets obtained from experimental studies examining the gonococcus grown under a variety of conditions and applied network analysis to infer the first gene coexpression network of N. gonorrhoeae. This network was then interrogated to identify transcripts of high centrality as well as to better define functional groups of genes that respond to certain environmental stimuli specifically related to infection. We also utilized the gonococcal network to expand our knowledge of regulatory pathways in this organism and to assign new putative categories to several proteins by using GBA analysis. This study has defined how gonococcal processes are related in this human pathogen and which of them have the potential to be important during human infection. This work will aid in the development of new antimicrobial treatment strategies, a critical gap that will need to be filled as N. gonorrhoeae continues to show resistance to a number of antibiotics.

## RESULTS

### Input data for network analysis.

**FIG 1:**
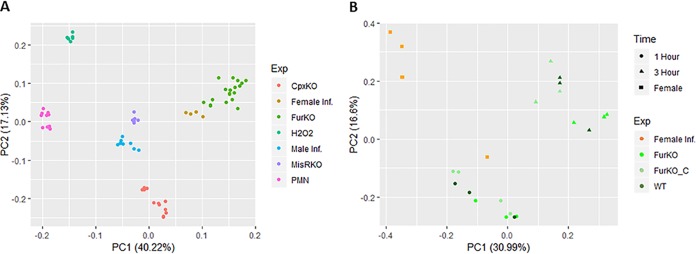
Principal-component analysis of conditions. (A) PCA analysis was carried out to define distances between all samples. Color indicates the experimental group, with the variability explained by the first and second principle components on the *x* and *y* axes, respectively. The group Cpx contains both *cpxA* and *cpxR* mutant data as well as the WT data from this experiment. The same grouping is carried out with *fur* and *misR* data, and the wild-type strain is included in each of these experimental clusters. For female and male infection, H_2_O_2_, and PMN exposure data, the control data sets are also included in the experimental group. (B) Specific analysis of several data sets that were tightly clustered in panel A. Two experiments are included in this graph: analysis of a *fur* mutant strain at 1 and 3 h after the addition of iron and analysis of N. gonorrhoeae during female genital tract infection. Colors indicate specific experiments and, for *fur* mutant experimental conditions, circles indicate 1 h after the addition of iron and triangles indicate 3 h after the addition of iron, while squares indicate infection of female genital tract.

We collected a total of 65 transcriptome sequencing (RNA-seq) data sets of the N. gonorrhoeae transcriptome during growth under a variety of conditions (see Table S1 in the supplemental material) (17–19, 22, 25, 26). These were either generated for this study or collected from the GEO database as publicly available data sets (see Materials and Methods). Gene expression values for all data are included in Table S3. RNA-seq data sets from these different studies were aligned to the N. gonorrhoeae FA1090 genome to determine gene expression levels in each of the samples. A subsequent principal-component analysis (PCA) showed that samples from the same experiment clustered strongly together (Fig. 1A) and that there was a large amount of transcriptomic variability between experiments. We observed significant similarity between samples collected from N. gonorrhoeae cultured *in vitro* under iron-deplete or -replete conditions, collected from N. gonorrhoeae strains lacking the Fur protein, and in samples obtained from cervicovaginal lavage specimens. Other groups have also reported variable gene expression results with gonococcal RNA extracted in different laboratories despite similar growth conditions. The observation that there are significant biological differences in the transcriptional responses among the data sets that we included is a strength of our analysis. Including data sets that induce changes in expression for a large number of different genes increases the chances of finding gene pairs that have a significant coexpression patterns and improves the robustness of our overall network.

10.1128/mSystems.00729-19.5 TABLE S1 Sample information for data used to infer network. Download Table S1, XLSX file, 0.02 MB. Copyright © 2020 McClure et al. 2020 McClure et al. This content is distributed under the terms of the Creative Commons Attribution 4.0 International license.

### Network inference.

We next used the 65 data sets to infer a network with the context likelihood of relatedness (CLR) program. We chose to use all 65 data sets, since gene coexpression networks are more robust and accurate if the maximum amount of RNA-seq data are included to infer them. This is because (i) a larger number of instances of expression for a given gene leads to a greater chance that a statistically significant instance of high mutual information will be found between this gene and another gene in the genome. Thus, variability between control conditions across laboratory groups is advantageous. (ii) A larger number of conditions allows for a greater number of genes that exhibit changes in expression under at least some of the conditions tested. This is critical for forming a robust and complete network. If a gene shows no change in expression across all the conditions tested, then there is essentially no expression pattern that emerges that can be linked to the expression patterns of others genes. Therefore, this nonresponsive gene will not be linked to any others and will not be included in the network. If this happens with many genes, as might be the case with a small number of conditions tested, then many genes will not be linked to others and the resulting network will be sparse. This study, by including as many conditions as possible, maximizes the chance that genes will show relevant changes in expression, be linked to other genes showing corresponding changes, and be included in the network, leading to a better and more robust network for downstream analysis.

**FIG 2:**
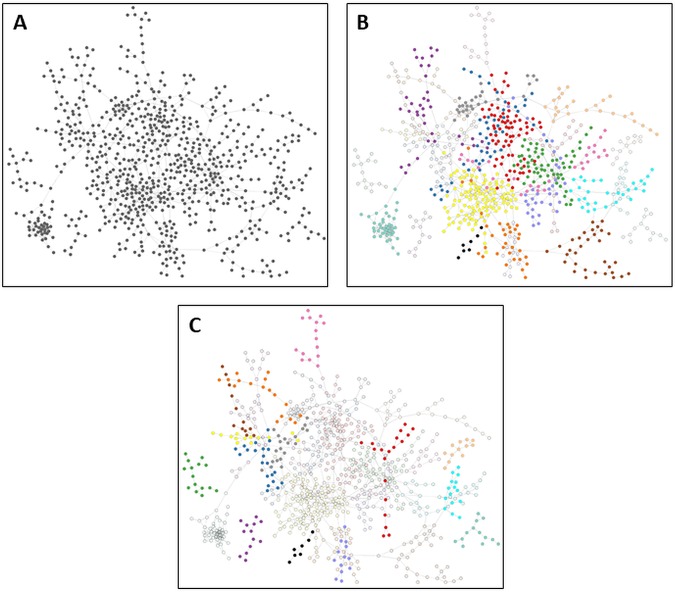
Network clustering of genes. (A) All transcriptomic data were examined using CLR to infer a network that links genes based on coexpression. Each gray circle represents an N. gonorrhoeae gene, and each line represents an instance of coexpression between a gene pair. Networks were viewed using Cytoscape, which attempts to cluster groups of highly linked genes together. (B) Genes in the network were grouped into 1 of 26 modules (groups of highly coexpressed genes). The 13 largest modules shown are distinguished by color. Genes that are faded are in smaller modules, while genes that are colored black were not grouped into any module. (C) The remaining 13 smaller modules shown are distinguished by color. Genes that are faded are in larger modules, and genes that are colored black were not grouped into any module.

Within the resulting network, nodes were represented by N. gonorrhoeae genes, and edges were represented by instances of high mutual information between genes. This network contained 1,118 N. gonorrhoeae genes (representing ∼56% of the gonococcal genome) with 1,499 edges between them (Fig. 2A). The node degree distribution was found to fit a power law with an *R*^2^ value of 0.903 (see Fig. S1), indicating that the network was scale free, a common characteristic of biological networks inferred for other systems (31), which will aid in analysis by helping to identify highly central nodes.

10.1128/mSystems.00729-19.1 FIG S1 Node degree distribution. Degree for nodes is shown on the *x* axis (log scale) and the number of nodes with that degree is shown on *y* axis (log scale). The degree distribution followed a power law with an *R*^2^ of 0.903 (shown). Download FIG S1, PDF file, 0.1 MB. Copyright © 2020 McClure et al. 2020 McClure et al. This content is distributed under the terms of the Creative Commons Attribution 4.0 International license.

A number of studies have found that genes that are of high centrality in coexpression networks are those that are critical for the growth, replication, and, in the case of human pathogens, infection (27, 28, 32). To identify such genes in N. gonorrhoeae, we next determined centrality values of both degree (the number of edges a node has with other nodes in the network) and betweenness (a measure of how many shortest paths through the network pass through the node in question) for each node in the network. The most highly connected nodes (those with the highest degree values) were tRNA genes. These had degree values that ranged from 7 to 17, with many tRNAs having degree values of at least 12. Most of the edges that tRNA genes had were to other tRNA genes, and, as described below, a large cluster of tRNAs was present in the network as a module of tightly connected nodes. The phenomenon of all, or nearly all, tRNA genes forming a tight cluster has been seen with other networks inferred for bacterial species (33). Aside from tRNA genes, other genes with high degree values included NGO0508, a phage-associated gene, NGO1506, an NTP pyrophosphohydrolase, NGO1818, RNA polymerase subunit alpha, NGO1741 and NGO1743, two NADH subunits, and NGO942, NGO1825 (*rplF*), and NGO1853 (*rplJ*), a 23s rRNA methyltransferase and two 50s ribosomal proteins, respectively. In addition to certain well-characterized genes, we also identified hypothetical proteins that had high degree values. These include NGO0745 and NGO1586. While the function of these genes is unknown, their highly connected position in the network suggests they have important roles and should be investigated further.

When examining betweenness we obtained somewhat different results, in that tRNA genes did not have high betweenness values. Instead, high betweenness genes included NGO1132, a phage-associated gene, two transporters, NGO1290 and NGO0446, a number of ribosomal protein genes, NGO1834 (*rpsS*), NGO1835 (*rplB*), and NGO1838 (*rplC*), and several hypothetical proteins. These hypothetical proteins include NGO0672, NGO1301, NGO1861, NGO2068 (a putative membrane protein), NGO1518, NGO0791 (containing a domain with lipoic acid-binding regulatory protein homology), and NGO0350 (containing a domain with hemolysin binding homology). Degree and betweenness values for all genes in the network are shown in Table S2. We also examined what processes in N. gonorrhoeae had the highest degree or betweenness values by averaging the centrality scores for all genes associated within a particular process. This showed that genes involved in carbohydrate metabolism, phage, and metabolism of amino acids and lipids had the highest betweenness values and genes involved in energy metabolism, translation, transcription, and tRNAs had the highest degree values (see Fig. S2).

10.1128/mSystems.00729-19.2 FIG S2 Average centrality values of all genes belonging to certain functions. (A) The average centrality value for all genes of the indicated functions in the network is shown. Functions are shown on the *x* axis and betweenness centrality is shown on the *y* axis. (B) The same analysis but examining degree centrality. Download FIG S2, PDF file, 0.4 MB. Copyright © 2020 McClure et al. 2020 McClure et al. This content is distributed under the terms of the Creative Commons Attribution 4.0 International license.

10.1128/mSystems.00729-19.6 TABLE S2 Centrality values for genes found in the network. Download Table S2, XLSX file, 0.04 MB. Copyright © 2020 McClure et al. 2020 McClure et al. This content is distributed under the terms of the Creative Commons Attribution 4.0 International license.

10.1128/mSystems.00729-19.7 TABLE S3 Annotation, centrality, module, and expression data for all genes. *^a^* NA indicates that the gene was not found in the main network cluster and therefore has no betweenness centrality. *^b^* NA indicates that the gene was not found in the main network cluster and therefore has no degree value. *^c^* NA indicates that the gene was not found in the network and there is in no module. A “0” indicates that the gene was in the network but was not clustered into any module. Download Table S3, XLSX file, 1.4 MB. Copyright © 2020 McClure et al. 2020 McClure et al. This content is distributed under the terms of the Creative Commons Attribution 4.0 International license.

### Module structure of networks.

We next examined the structure of the network in more detail by grouping genes in the network into discrete modules, defined as highly connected clusters of genes. This was performed by assigning genes in the network to modules such that there were more edges linking genes in the same module than edges linking genes in two different modules. This analysis identified 26 different modules, with between 12 and 109 genes per module (Fig. 2B and C and Table 1). Genes of a related function are often linked in a gene coexpression network, and because of this, genes of similar functions are often clustered into the same module. To explore this further, we carried out functional enrichment on each of the modules in the network to determine if certain functions were more highly represented in a particular module than in the genome as a whole. This analysis found that in the majority of modules, at least one function was statistically significantly enriched, with several modules being enriched for multiple functions.

**TABLE 1:**
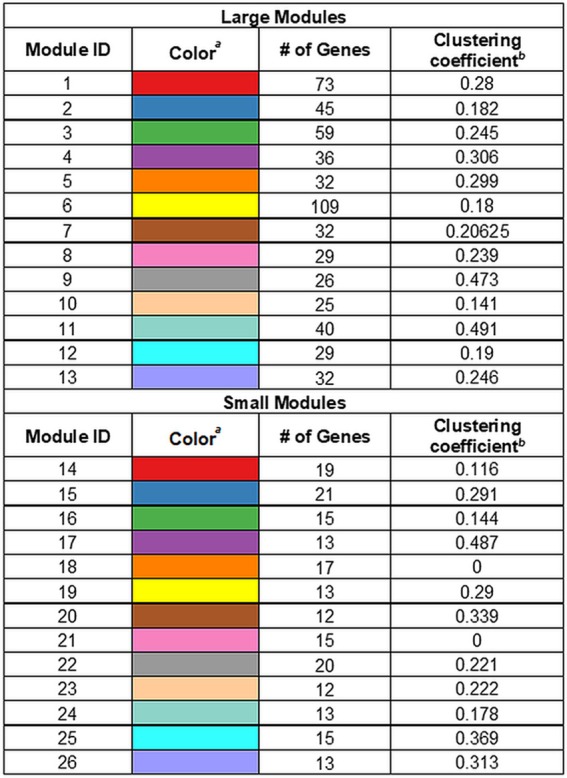
Network modules

a The colors of the modules as depicted in Fig. 2 are shown.

b The clustering coefficients of the modules are shown, a measure of the ratio of node triplets/edges in the module.

The largest module was module 6, with genes involved in host interactions in this module, including genes encoding toxin-antitoxin systems (NGO1067 and NGO1068 [*mafA* and *mafB*]) and proteins involved in antibiotic resistance (Mtr pump). Module 7 was enriched for a number of processes, including environmental information processing, containing several transporters. Module 8 was enriched for amino acid metabolism processes and included genes such NGO1541 (*murE*), NGO1667 (*dapD*), and NGO1808. Module 10 was strongly enriched for genes involved in genetic information processing such as translation and transcription and included genes encoding several ribosomal proteins and two RNA polymerase subunits. Module 13 contained a number of pilin-related genes (*pilI*, *pilJ*, and *pilH*) and was also enriched for energy metabolism processes. Module 17 was enriched for phage-associated genes, and module 34 was enriched for lipid metabolism, containing NGO1763 and NGO2163 (*fabG*) (Table 2).

**TABLE 2:** Functional enrichment of modules

Module ID^*a*^	Enriched function	Percentage in:^*b*^		Ratio^*c*^	*P* value^*d*^
		Module	Genome		
1	Energy metabolism	0.233	0.035	6.6	>0.001
2	Nucleotide metabolism	0.089	0.022	4.0	0.020
5	Cellular processes/organismal systems/human disease	0.250	0.111	2.2	0.023
5	Hypothetical	0.563	0.368	1.5	0.027
7	Energy metabolism	0.188	0.035	5.3	0.001
7	Environmental information processing	0.125	0.032	4.0	0.020
7	Enzyme families	0.063	0.010	6.3	0.046
8	Amino acid metabolism	0.138	0.045	3.1	0.043
9	Genetic information processing	0.615	0.158	3.9	>0.001
10	Genetic information processing	0.360	0.158	2.3	0.012
11	tRNA	0.875	0.026	33.3	>0.001
12	Genetic information processing	0.552	0.158	3.5	>0.001
13	Energy metabolism	0.188	0.035	5.3	0.001
14	Phage associated	0.263	0.064	4.1	0.006
16	Genetic information processing	0.533	0.158	3.4	0.001
17	rRNA	0.154	0.003	58.6	0.001
17	Phage associated	0.385	0.064	6.0	0.001
22	Amino acid metabolism	0.250	0.045	5.5	0.002
25	Energy metabolism	0.200	0.035	5.7	0.015
34	Lipid metabolism	0.154	0.011	13.9	0.010

a ID, identifier.

b The percentage of N. gonorrhoeae genes with the indicated function.

c The ratio between the in-module percentage and the in-genome percentage.

d The *P* value of the enrichment using Fisher’s exact test.

We next examined linkages between modules to better understand how pathways and processes in N. gonorrhoeae were related. Many of the modules were large and were highly connected to several other genes and modules within the network, making specific connections more difficult to interrogate. However, modules 7, 9, 10, and 12 occupied positions on the periphery of the network and had fewer connections with other modules. Module 7 was enriched specifically for environmental information processing and contained genes for iron transporters (NGO0215, NGO0216, and NGO0217 [*fbpA*, *-B*, and -*C*]), a polyamine transporter (NGO1494), and a twin-arginine protein transporter (NGO0182). A number of cytochrome genes were also grouped into this module, including NGO2030, NGO1328, and NGO1371. This module was also highly segregated from the rest of the network, with only 3 genes connecting it to modules 12 and 5. One of these edges passed through the *agrA* gene of module 7 and linked it to the *mafB* gene of module 5. The position of the *mafB* genes and *agr* genes at the confluence of two modules suggests their possible roles in processes that are found in each module. This includes the processes described above for module 7 as well as infection-related processes found in module 5 (several pilin and opacity proteins).

### Response of genes in the network to specific conditions.

**FIG 3:**
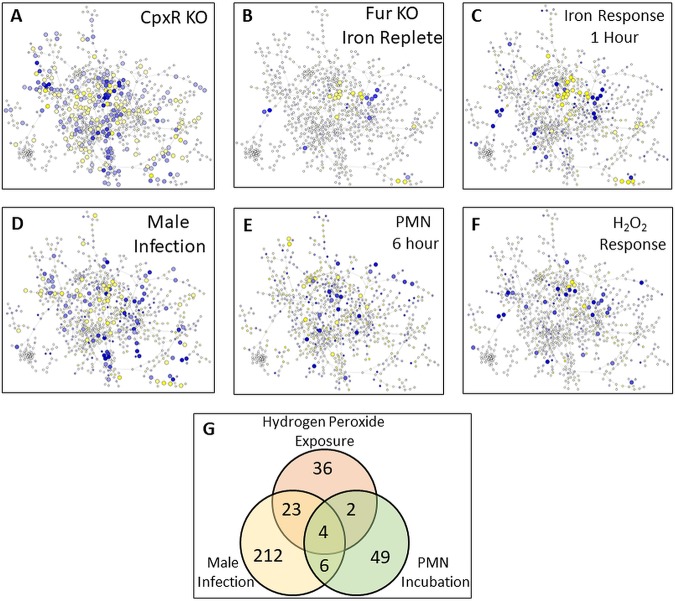
Response of the gonococcal network to specific conditions. Genes within the network responding to specific conditions are shown. For each condition, expression data were compared between treatment (either a mutant strain or environmental perturbation) and a control (either a wild-type strain or an environmental control). Genes showing a >2-fold change in expression with an adjusted *P* value of <0.05 are shown as larger nodes in the network. Large yellow nodes indicate genes that were expressed at higher levels under control versus treatment conditions, and large blue nodes indicate genes that were expressed at lower levels under control versus treatment conditions. The color shading of the node indicates the strength of the response: darker nodes show a stronger response, either increased or decreased. (A) Analysis of *cpxR* mutant strain. (B) Analysis of *fur* mutant strain under iron-replete conditions. (C) Analysis of the gonococcal response 1 h after the addition of iron. (D) Analysis of infection of the male genital tract. (E) Analysis of incubation in PMNs after 6 h. (F) Analysis of response to H_2_O_2_ incubation. (G) A Venn diagram showing the number of differentially expressed genes for three oxidative stress-related conditions (hydrogen peroxide exposure, infection of the male genital tract, and incubation in PMNs) and the overlap in differentially expressed gene (DEG) number for each pair of conditions and for all three conditions.

The network we inferred here included a large amount of transcriptomic data from several different growth conditions. While the results above are from an analysis of all data, we also wanted to determine how parts of the network responded to six specific conditions (Fig. 3). The first was the response of N. gonorrhoeae to loss of the CpxR regulator (18). A comparison of the transcriptome of the wild-type (WT) strain to that of a *cpxR* mutant strain identified a very large number of genes showing differential expression (242 genes), with these genes being spread throughout the network. In contrast, a comparison of the transcriptome of the wild-type strain to that of a *fur* mutant showed a much smaller and focused response, with few very tight clusters of genes (24) in the network showing differential regulation. These Fur-responsive genes were found mainly in module 1, enriched for energy metabolism, and module 14, enriched for phage-associated processes. Examining the network response to iron (in a wild-type strain containing a functional Fur protein), we found a slightly larger response (45 genes) and, as expected, a large overlap with the network response to a *fur* mutant strain. Most of these genes responding to iron fell into modules 1 and 14, but module 7 was also represented (enriched for energy metabolism and environmental information processing) as well as module 6.

We also looked specifically at gonococcal transcriptomes derived from urethral swabs of infected men and compared them to transcriptomes of the same strains cultured *in vitro*. With this comparison, several genes in different parts of the network were differentially regulated (103 genes). The response of N. gonorrhoeae to PMNs was less robust and widespread than the response to infection of the male genital tract (30 versus 103 genes, respectively). Parts of the network that responded to incubation with PMNs showed strong overlap with module 10, which contained a number of translation and transcription genes as well as several regulatory proteins. Finally, we also included data examining the response of N. gonorrhoeae to incubation with hydrogen peroxide. Gonococcal genes responding to incubation with hydrogen peroxide included several members of modules 3 and 14, with the latter being functionally enriched for phage-associated genes. The function of most of these gonococcal phage-associated genes is unknown, but previous studies have assigned a role for some (such as NGO1013) to infection (34). There was relatively little overlap among genes that were differentially expressed as a function of incubation with PMNs and those that were differentially expressed as a function of either infection of the male genital tract or exposure to H_2_O_2_. However, there was significant overlap among differentially expressed genes that were expressed as a function of H_2_O_2_ exposure and infection of the male genital tract, with 27/64 (∼42%) of the genes responding to H_2_O_2_ exposure also responding to infection of the male genital tract. These included several genes encoding energy metabolism processes, including cytochrome *c*, as well as molecular chaperone stress response proteins. Four genes were found to be regulated under all three conditions, two hypothetical proteins (NGO0637 and NGO1948), fumarate hydratase (NGO1029 [*fumC*]), and thioredoxin/methionine sulfoxide reductase (NGO2059). Responses of the network to other conditions that were part of the data set are shown in Fig. S3, and lists of differentially expressed genes for all conditions are shown in Table S4. We also examined the average responses of genes in each module to gain information on how whole modules, and the functions they were enriched for, responded to specific conditions (see Fig. S4).

10.1128/mSystems.00729-19.3 FIG S3 Response of network to specific conditions. Genes within the network responding to specific conditions are shown. For each condition, expression data were compared between treatment (either a mutant or environmental perturbation) and a control (either a wild-type strain or an environmental control). Genes showing a ≥2-fold change in expression with an adjusted *P* value of ≤0.05 are shown as larger nodes in the network. Large yellow nodes indicate genes that are expressed at higher levels under control than under treatment conditions and large blue nodes indicate genes that are expressed at lower levels under control than under treatment conditions. The color shading of the node indicates the strength of the response: darker nodes show a stronger response, either increased or decreased. (Top left) Analysis of *cpxA* mutant strain. (Top middle) Analysis of infection of the female genital tract. (Top right) Analysis of incubation in PMNs after 3 h. (Bottom left) Analysis of *fur* mutant strain under iron-deplete conditions. (Bottom middle) Analysis *misR* mutant strain. (Bottom right) Analysis of the gonococcal response 3 h after the addition of iron. Download FIG S3, PDF file, 0.5 MB. Copyright © 2020 McClure et al. 2020 McClure et al. This content is distributed under the terms of the Creative Commons Attribution 4.0 International license.

10.1128/mSystems.00729-19.4 FIG S4 Response of modules to specific conditions. The median expression level for all genes within each module was normalized to the mean of the row. The ratios are shown across all conditions (using the mean from all biological replicates). Blue color indicates lower expression for that condition than the mean and yellow indicates higher expression. Hierarchical clustering was applied to modules to group those that had a similar expression profiles across conditions. Download FIG S4, PDF file, 0.02 MB. Copyright © 2020 McClure et al. 2020 McClure et al. This content is distributed under the terms of the Creative Commons Attribution 4.0 International license.

10.1128/mSystems.00729-19.8 TABLE S4 Fold change data for all genes comparing perturbations to controls. *^a^* Whether the gene is a differentially expressed gene (DEG) for the comparison, defined as a *P* value of <0.05 and a log2 fold change of <−1 or >1, is indicated. Download Table S4, XLSX file, 0.6 MB. Copyright © 2020 McClure et al. 2020 McClure et al. This content is distributed under the terms of the Creative Commons Attribution 4.0 International license.

### Network analysis of gonococcal genes involved in infection.

**FIG 4:**
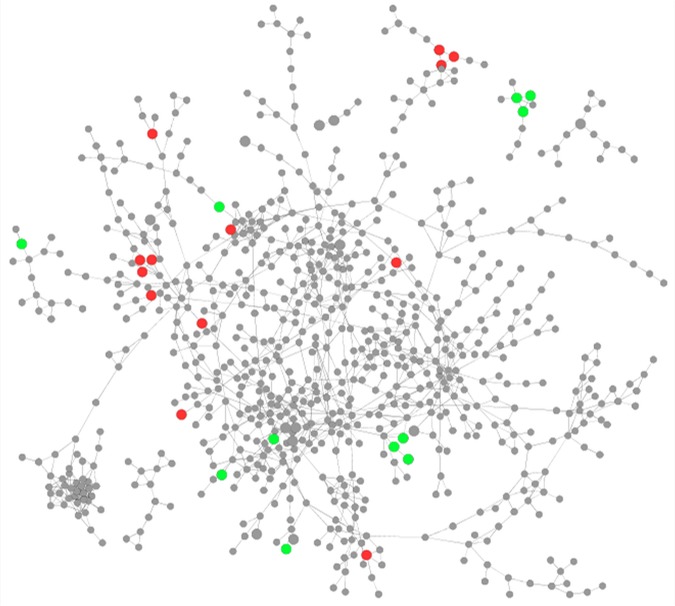
Network position of infection-related genes. Network with pilin-related genes (green circles) and stress-related genes (red circles) highlighted.

We next used the network to examine the location and surrounding network neighborhood of genes that are known to have a role in gonococcal infection. We defined these as genes that are involved in pilin (involved in attachment to host cells) or host (adhesins and antimicrobial-resistant proteins) interactions (Fig. 4). Several of these genes were clustered together into coexpressed pairs, triads, or tetrads of genes, again demonstrating how clustering genes based on coexpression also acts to cluster based on function. We next examined clusters of infection-related genes to examine their network neighborhood more closely and determine which genes they were connected with. The first cluster consisted of a pair of genes, both involved in antibiotic resistance and efflux of antibiotics (NGO1363 and NGO1364). These genes were connected to two other genes, both of which were phage-associated genes (NGO1625 and NGO1626). A second cluster consisted of three genes involved in pilin biogenesis (NGO0096 to NGO0098 [*pilO*, *pilN*, and *pilM*]). These genes were connected to a hypothetical protein (NGO0549) as well as a thioredoxin reductase gene (NGO0580) and an AraC family regulator (NGO0990).

### Using network analysis to explore regulatory pathways in N. gonorrhoeae.

**FIG 5:**
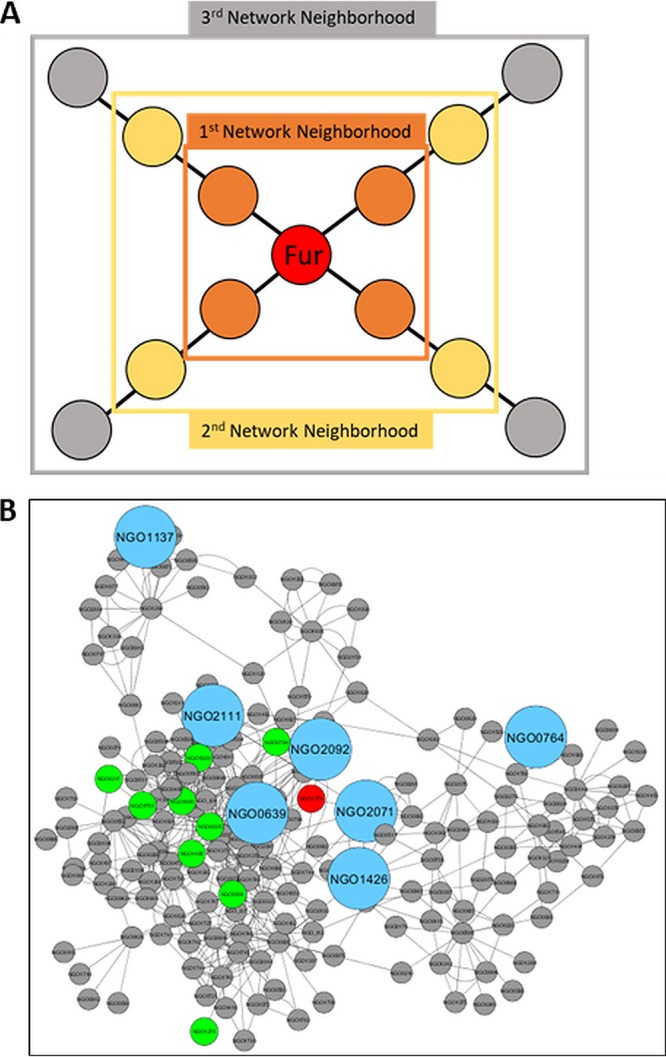
Network neighborhood of Fur. (A) Schematic of how network neighborhood is defined. (B) The third network neighborhood of Fur in a network inferred using GENIE3. The *fur* gene is shown as a large red node, with known targets of Fur shown as larger green nodes and putative new targets of Fur, those that contain a Fur binding site and are within the network neighborhood, shown as much larger blue nodes.

Previous studies have used network analysis to expand knowledge of regulatory pathways in bacteria by looking for edges that link regulators and putative targets based on coexpression (35). To apply this to N. gonorrhoeae, we next focused on using the gene coexpression network to expand the regulon of a single regulatory protein, Fur (36). One study compared the ability of several network inference methods to predict known targets of regulators in Escherichia coli and found that a random forest method, GENIE3 (37), was the most accurate for this specific approach (38). To that end, we inferred a new network from our data using GENIE3 and collected the 3rd-order network neighborhood of Fur. This consisted of genes that were linked to Fur through no more than two additional genes (Fig. 5A). This network neighborhood contained 179 genes (of 1,786 in the GENIE3 network) as well as 9/23 of the known targets of Fur (Fig. 5B). The observation that the GENIE3 network was not able to identify all targets of Fur is likely due to some targets not responding under the conditions that were included in this study. It is also possible that the activation of Fur and the impact on a target gene’s expression may be posttranscriptional and thus not captured in the transcriptional data used to infer the network. Despite the observation that the GENIE3 network was not able to identify all targets of Fur, it did identify ∼40% of the known targets among a subnetwork containing a relatively small number of genes. There was a 0.012% possibility of selecting these Fur targets by chance if 179 genes were randomly chosen from the network. This demonstrated that our network neighborhood was able to accurately gather several known Fur targets in a statistically significant way. We then examined the promoter regions of all 179 genes in the neighborhood to determine if they contained a Fur binding site, defined as GATAATGATAATCATTATC (15). We found that several targets of Fur in this network neighborhood that were known to be directly regulated (i.e., Fur binds to their promoter regions) contained Fur binding sites by our analysis. These included NGO0904 (15), *aniA* (NGO1276), and *tbpB* (NG1496). We also found an additional seven genes within this network neighborhood that contained Fur binding sites in their promoter regions (Fig. 5B) but, to date, have not yet been shown to be targets of Fur. Several of these genes were also regulated via iron based on a previous analysis (17), including NGO0639, NGO2071, NGO2092, and NGO2111. In addition, several of these same genes were also regulated as a function of the absence of Fur, including NGO0639, NGO2092, and NGO2111 (17).

### Using network analysis to assign putative categories to genes.

**FIG 6:**
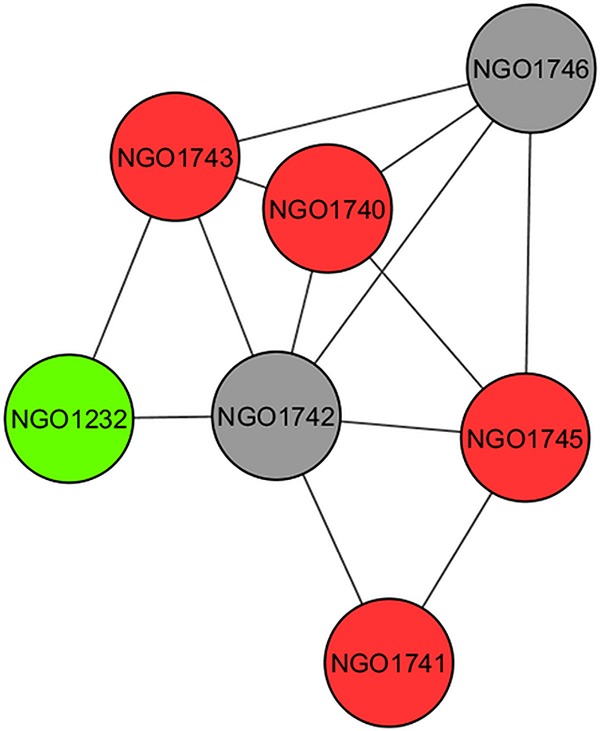
GBA analysis of hypothetical proteins. NGO1742, encoding a hypothetical protein, within the network was extracted along with the six additional genes that it has an edge with. Red genes are those that are involved with energy metabolism. The green gene is involved in DNA metabolism, and the gray gene is an additional hypothetical.

Functional enrichment of modules has shown that genes that are linked in the network are often of the same or similar function. Because of this, network structure can be used to assign putative categories to uncharacterized genes, a process termed guilt by association (GBA). This approach has been used with other species to predict functions of unknown genes based on the edges they have with well-characterized genes in the network (30). As an example, an unknown gene in N. gonorrhoeae, NGO1742, has edges with six other genes, four of which are involved in energy metabolism (Fig. 6). As genes of similar function are linked in the network, this observation suggests that this unknown gene may also be involved in energy metabolism. We then tested GBA further by determining how accurate the approach was by looking at how well it could predict the functional category of well-characterized genes of N. gonorrhoeae. Of eight genes that we tested with this approach, six were found to have the same function by GBA analysis as their known function.

After demonstrating the utility of this approach, we applied it to all N. gonorrhoeae genes as a way to expand our knowledge of functional categories of gonococcal genes. Using the definitions for an assigned category (see Materials and Methods), our initial analysis was focused on N. gonorrhoeae genes that had an assigned category by GBA that matched their known category in KEGG. We then looked within this list of genes to see if any of the genes had an additional, as yet unknown, category assigned by GBA that had not yet been assigned by KEGG. The ability of GBA analysis to identify the known category of a gene served as a way to confirm the accuracy of the process on a gene-by-gene basis. Using this approach, we were able to define additional new categories for 26 genes (Table 3). These categories included genetic information processing, carbohydrate metabolism, cellular processes/organismal systems/human disease, phage associated, energy metabolism, and amino acid metabolism. Note that this new category is not meant to replace the old category but rather to supplement it. As many genes may be involved in more than one category, this allowed us to expand our knowledge of N. gonorrhoeae gene functions.

**TABLE 3:** GBA analysis of N. gonorrhoeae genes

Gene	Category		*P* value^*b*^
	Known^*a*^	Newly assigned through GBA	
NGO0034	Carbohydrate metabolism	Genetic information processing	0.036
NGO0174	Genetic information processing	Carbohydrate metabolism	0.013
NGO0214^*c*^	Carbohydrate metabolism	Amino acid metabolism	0.008
NGO0214^*c*^	Carbohydrate metabolism	Genetic information processing	0.004
NGO0256	Genetic information processing	Phage associated	0.001
NGO0351	Genetic information processing	Carbohydrate metabolism	0.013
NGO0474	Phage associated	rRNA	0.022
NGO0506	Phage associated	Genetic information processing	0.016
NGO0711	Carbohydrate metabolism	Genetic information processing	0.002
NGO0719	Carbohydrate metabolism	Genetic information processing	0.004
NGO0890	Carbohydrate metabolism	Genetic information processing	0.036
NGO0899	Genetic information processing	Carbohydrate metabolism	0.013
NGO1129	Phage associated	rRNA	0.016
NGO1130	Phage associated	rRNA	0.016
NGO1325	Carbohydrate metabolism	Genetic information processing	0.016
NGO1616	Phage associated	rRNA	0.022
NGO1626	Phage associated	Energy metabolism	0.006
NGO1629	Phage associated	Cellular processes/organismal systems/human disease	0.000
NGO1809	Genetic information processing	Carbohydrate metabolism	0.001
NGO1823	Genetic information processing	Carbohydrate metabolism	0.013
NGO1831	Genetic information processing	Carbohydrate metabolism	0.005
NGO1836	Genetic information processing	Energy metabolism	0.006
NGO2030	Energy metabolism	Cellular processes/organismal systems/human disease	0.015
NGO2148	Energy metabolism	Genetic information processing	0.004
NGO2151	Energy metabolism	Genetic information processing	0.000
NGO2156	Carbohydrate metabolism	Genetic information processing	0.016

a The known category of the N. gonorrhoeae gene according to KEGG.

b The statistical enrichment of the function found by GBA analysis using Fisher’s exact test.

c Two categories were found with GBA for NGO0214, and so it is included in the table twice.

## DISCUSSION

Gonorrhea remains a high-morbidity disease within the United States and worldwide. The increased prevalence of gonorrhea combined with the rising number of antibiotic-resistant N. gonorrhoeae strains speaks to the need for a renewed focus on developing treatment strategies that target new pathways and processes in this organism. Here, we inferred and interrogated the first N. gonorrhoeae gene coexpression network to define (i) how processes are related and respond to specific conditions encountered during infection, (ii) which genes occupy positions of central importance, (iii) how regulatory pathways are organized, and (iv) additional functions for several genes in N. gonorrhoeae.

Within this data set were transcriptomic experiments resulting from changes to the growth environment of a N. gonorrhoeae wild-type strain (samples 13 to 49 and 56 to 65) (see Table S1 in the supplemental material) and changes to the genetic background of N. gonorrhoeae (knockout strains) (samples 1 to 12, 17 to 35, and 50 to 55) (Table S1). Other studies have found that transcriptomic data sets resulting from changes in growth conditions of the system under analysis are more valuable for network inference than data sets resulting from genetic perturbations (35, 39). The reasoning for this is unclear but is likely due to the fact that an environmental condition will induce changes in a greater number of genes than a genetic perturbation. As an example, studies of iron and Fur regulation in N. gonorrhoeae have found that while only 54 genes respond to a lack of the Fur protein (a genetic perturbation), 158 genes responded to changes in iron in the growth media (an environmental perturbation) (17). As transcriptomic data for N. gonorrhoeae becomes more available in the coming years, it may be beneficial to infer networks made primarily, if not completely, from transcriptomes resulting from environmental changes rather than genetic perturbations. It should be noted that among our data sets, environmental perturbations represent the majority of conditions analyzed.

The gonococcus expresses several genes that are essential to host cell interactions that have the potential to be new targets for drug development. Network analysis allows for targeting those genes that occupy the most central position in gonococcal virulence pathways, such that inhibition of these pathways will impact the ability of this pathogen to colonize and survive in the human host environment. We also used GBA analysis to expand our knowledge of gene category and function in N. gonorrhoeae. When using GBA to assign functions to uncharacterized genes, it should be emphasized that GBA results are a piece of data that must be viewed in the context of other information when trying to determine gene function. GBA results are not definitive categories for genes, but they do provide a specific possibility that can then be tested experimentally. In addition, GBA results could be improved by (i) adding more data to the network to make it more robust and (ii) focusing on certain experiments to focus the network toward specific functions. For example, if host-responsive genes are of specific interest, then a network could be built from only RNA derived from host-N. gonorrhoeae interaction experiments. Our goal here was to build a broad network, but a more focused network could also be of use in future studies.

Grouping genes in the gonococcal network based on coexpression allowed us to cluster genes with similar responses to specific conditions. As many of the conditions used to infer the network were related to human mucosal infection, network analysis allowed us to define how particular pathways responded to these infection-related conditions as well as highlight overlap between functions responding to multiple conditions. For example, the gonococcal response to PMN incubation resulted in differential expression of several genes that overlapped with modules enriched for genes of N. gonorrhoeae involved in protein production and growth, including several ribosomal proteins (L17, L19, L27, L33, RNA polymerases [NGO1850 and NGO1851 {*rpoB*}], and cytochrome *c*_1_ [NGO2031]). While engulfment by PMNs is a stress condition encountered by N. gonorrhoeae during human infection, gonococci can survive and replicate under these conditions (40). The overlap of genes responding to replication aspects of N. gonorrhoeae and PMN response in our network likely reflects gonococcal survival in PMNs. We also observed the differential expression of an AsnC-type regulator (NGO1294 [*lrp*]) during growth in PMNs. NGO1294 was previously demonstrated to respond to N. gonorrhoeae exposure to oxidative stress (41, 42). It is important to note that the identification of clusters responding to specific pathways (translation/transcription) and to a specific condition (PMN incubation) is only possible once a network of the type we show here has been built. These conclusions would be more difficult to draw when examining lists of up- or downregulated genes only. Organizing genes into a network of coexpression allows for new conclusions to be made based on the resulting network structure.

One of the original purposes for network analysis was to link regulatory proteins and potential targets (35). We have also taken this approach in the present study by expanding the regulon of Fur, a global regulator with a critical role in gonococcal pathogenesis. By looking for edges linking Fur to other genes, we identified a list of new putative targets. This list was improved by cross-referencing those targets that had a known Fur binding site in their promoter regions. This resulted in the identification of gonococcal genes known to be involved in iron metabolism, including *erpA* (NGO1426) and *fetA* (NGO2092). Expression of the *erpA* gene has been reported to respond to growth under anaerobic conditions (23). These results suggest that Fur controls the expression of oxygen-responsive genes. The female genital tract, an environment in which control of Fur is critical for iron acquisition by the gonococcus, is also an oxygen-deplete environment (43). Other studies have suggested that *fetA* is a target of Fur, an observation which was corroborated in this study (44). Several hypothetical proteins were also identified among the new putative targets of Fur, including NGO2071 and NGO2111. It is important to note that the network did not identify all known targets of Fur, and thus it is likely that additional targets remain unidentified in our GENIE3 network. The inclusion of more data, specifically, transcriptomic data centered on environmental conditions that known target genes respond to, and a reinference of the network could improve the network’s ability to identify known Fur targets. Alternatively, applying several different network inference methods (GENIE3, CLR, Pearson, etc.) and using a voting metric for genes highly linked to Fur could be used to identify additional targets. Collectively, these results not only expanded the gene repertoire of the Fur regulon but also demonstrated the power of network analysis in expanding our understanding of gonococcal regulatory pathways.

In conclusion, we present the first gene coexpression network of N. gonorrhoeae and demonstrate how it can be used to define processes and regulatory pathways in this organism that are central to infection. Not only will the data presented here inform new hypotheses, but the network itself will act as a resource for the community as other scientists query the location of genes of interest in the network and what genes they are connected to. Network inference has emerged over the past decade as a powerful tool for a high-level, but gene-specific, view of a number of systems. Its application here to N. gonorrhoeae identifies centrally important genes in this organism that can be the target of future efforts to develop new drug treatments. It also greatly enhances our knowledge of the putative functions of uncharacterized genes in this pathogen and expands the role of regulatory proteins that are critical for infection. Antibiotic resistance emerging in N. gonorrhoeae is only likely to increase in the coming decades, and new treatment strategies are critically needed. The data and conclusions presented here can be mined to develop these strategies and combat this STI with new and more-targeted drugs that treat this disease and reduce its morbidity.

## MATERIALS AND METHODS

### Collection of transcriptomic data.

Transcriptomic data used for network analysis were either already available in the Gene Expression Omnibus (GEO) database or generated by our group (see Table S1 in the supplemental material). Data used in this study and available on GEO included samples 1 to 26 in Table S1 (*cpxA* and *cpxR* mutant strains, cervicovaginal lavage specimens, and analysis of N. gonorrhoeae wild-type, *fur* mutant, and complemented *fur* mutant strains 1 h after the addition or removal of iron) and samples 36 to 55 (hydrogen peroxide treatment, *misR* mutant strain, and urethral swab specimens). Additional new data sets used in this study were obtained from N. gonorrhoeae during incubation with human PMNs and N. gonorrhoeae wild-type, *fur* mutant, and complemented *fur* mutant strains grown *in vitro* (3 h postaddition or depletion of iron, as opposed to 1 h as mentioned above). Overall, we collected and analyzed data from 26 different conditions with between 1 and 4 biological replicates per condition for a total of 65 individual data sets. Table S1 describes the conditions, references, and GEO accession numbers for the publicly available data sets as well as preprocessing steps and alignment results for all data. Methods for growth, RNA isolation, and sequencing for N. gonorrhoeae wild-type, *fur* mutant, and complemented *fur* mutant strains grown *in vitro* for 3 h after the addition and removal of iron are identical to those used to examine a 1-h time point and were described previously (23). For incubation of N. gonorrhoeae in PMNs, these were isolated from venous blood collected from healthy volunteers, as previously described (45). Written consent was obtained from each volunteer in accordance with a protocol approved by the Institutional Review Board for Human Subjects at the University of Iowa. Briefly, heparinized blood was collected, and PMNs were isolated using dextran sedimentation followed by density gradient separation on Ficoll-Paque Plus. After hypotonic lysis of erythrocytes, PMNs were resuspended in Hanks balanced salt solution (HBSS) without divalent cations containing 20 mM HEPES and 1% human serum albumin and adjusted to 20 × 10^6^ cells/ml. N. gonorrhoeae 1291 wild-type (from M. A. Apicella’s laboratory) was fed to PMNs at 10:1 multiplicity of infection (MOI) for 10 min followed by removal of unbound bacteria and then incubations at 37°C for 10 min, 180 min, and 360 min. RNA was isolated using TRIzol reagent, followed by rRNA depletion with Ribo-Zero bacteria rRNA removal kit (Illumina). RNA samples were analyzed using an Illumina platform at the Iowa Institute of Human Genetics (IIHG)-Genomics division.

### Alignment and normalization of sequencing data to the N. gonorrhoeae genome.

All raw data were in the form of fastq files. These were first analyzed with FastQC to determine if trimming was necessary either to remove adaptor sequences or to remove nucleotides of low quality. Certain samples were trimmed of adaptor sequences (Table S1), and all nucleotides with a Phred quality score of <25 were removed from all reads. Trimmed fastq files were then aligned to the genome of N. gonorrhoeae FA1090 (NCBI NC_002946.2) using the Burrows-Wheeler aligner (BWA; bwa-mem command) with default settings (46). The resulting .sam files were then used with the .gtf file for N. gonorrhoeae FA1090 and HTseq (47), with default settings, to determine raw counts for each gene in the gonococcal genome. All transcriptomic data were aligned to a single strain, FA1090, so that each gene would be represented by a single node and not by multiple nodes, each representing a homologue of the same gene found in multiple N. gonorrhoeae strains. It should be noted that this analysis can be repeated after alignment to other gonococcal genomes of interest, such as those that contain the gonococcal genetic island (GGI). Raw counts were then normalized using DESeq2 (48), and fold changes comparing conditions to their matched controls were also calculated using DESeq2. Detailed descriptions of computational methods are given in Text S1.

10.1128/mSystems.00729-19.9 TEXT S1 Detailed computational methods used to align and normalize the transcriptomic data as well as infer and analyze the network. Download Text S1, DOCX file, 0.02 MB. Copyright © 2020 McClure et al. 2020 McClure et al. This content is distributed under the terms of the Creative Commons Attribution 4.0 International license.

### Network inference.

Once we obtained normalized gene expression levels, we inferred a network of all genes using CLR (35), a version of which is part of the MINET package in R (49). R is a freely available programming language and is a very common tool used in computational biology with a large number of users and support. CLR outputs a matrix of Z-scores of mutual information values for each gene pair, and we inferred several networks of different sizes by varying the cutoff Z-score we used to define an edge in the network. Nine different networks were inferred containing between 10,000 and 1,000 edges with Z-score cutoffs of between ∼5.56 and ∼8.18. After examining all nine networks, we selected a network of 1,500 edges (with a Z-score cutoff of 7.701072) for further analysis. This network was chosen as it had significant structure (clusters of distinct tightly coexpressed genes) and contained a greater number of genes than a network of 1,000 edges. Detailed descriptions of computational methods are given in Text S1. A file containing the network in .sif format listing all the edges is also included in Text S2.

10.1128/mSystems.00729-19.10 TEXT S2 A file in .sif format that can be imported into Cytoscape so that the network can be easily browsed. Download Text S2, TXT file, 0.03 MB. Copyright © 2020 McClure et al. 2020 McClure et al. This content is distributed under the terms of the Creative Commons Attribution 4.0 International license.

### Network interrogation.

Networks were viewed using Cytoscape (50), and modules were detected using the igraph package in R (51). The clustering coefficient for modules is defined as the average clustering coefficient of all nodes in the module. This is the ratio of the number of closed three-node triangles that a node was a part of to the number of open three-node triangles that a node was a part of and was calculated using Cytoscape. Functional enrichment was carried out on genes grouped into modules as described previously (26). Centrality analyses were carried out only on the main network cluster to avoid inflated betweenness values for small unconnected groups of nodes.

### Fur regulon analysis and GBA analysis.

To identify new targets of Fur, we collected promoter regions for each gene in the 3rd-degree network neighborhood of Fur. Promoter regions were defined as the 250 bp upstream of the start codon. Each of these sequences was examined with the Find Individual Motif Occurrences (FIMO) program (52) to identify those sequences that contained a Fur binding site, defined as GATAATGATAATCATTATC (15). We then highlighted genes in the network neighborhood of Fur that also contained a Fur binding site to identify potential new targets of Fur. To carry out GBA analysis, we collected the 75 genes that had the highest coexpression values (Z-scores) with a given protein. We then carried out functional enrichment on that group of genes to determine if any known functions were found (33). A putative category was assigned to a protein (i) if at least 10% of the genes in this 75-gene data set were assigned to that category, (ii) if this percentage was higher than the percentage of genes of this same category in the genome as a whole, and (iii) if this increase was significant (*P* value < 0.05) using Fisher’s exact test.

### Data availability.

Transcriptomic data from WT (strain F62), *fur* mutant, and completed *fur* mutant strains after a 3-h incubation under both iron-replete and -deplete conditions have been deposited in GEO under accession number GSE143480. Transcriptomic data from incubation of N. gonorrhoeae (strain 1291) in PMNs have been deposited in GEO under accession number GSE143553. A .tar file containing the .cys file of the network, to be opened in Cytoscape, is available at https://datahub.pnnl.gov/datahub/project/25.
