# Supplementary figures and images for: Global Network Analysis of Neisseria gonorrhoeae Identifies Coordination between Pathways, Processes, and Regulators Expressed during Human Infection
Source: mSystems. 2020 Feb 4;5(1):e00729-19. doi: 10.1128/mSystems.00729-19 (PMC7002116; doi:10.1128/mSystems.00729-19)

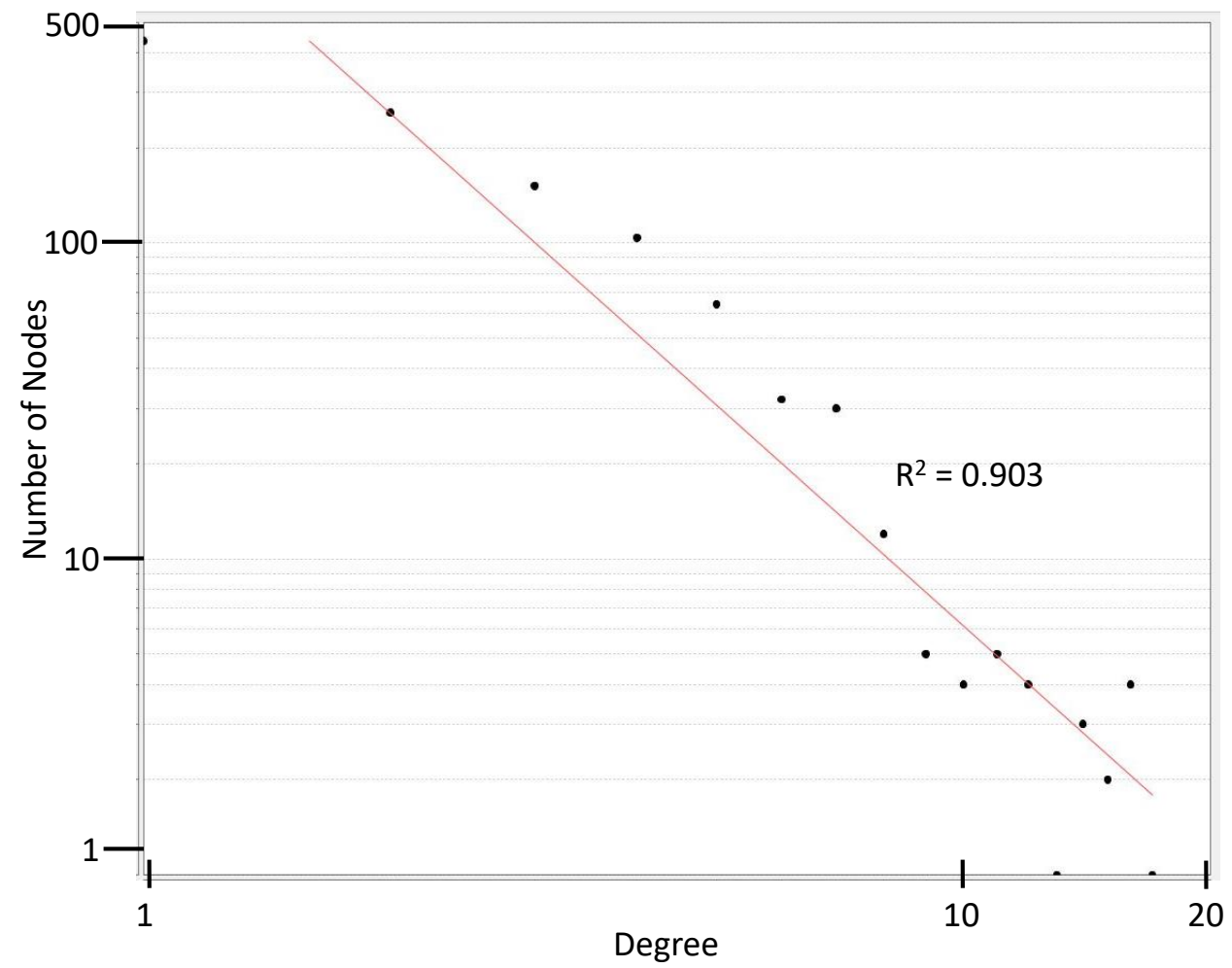

Supplement: FIG S1 [file mSystems.00729-19-sf001.pdf]

**A**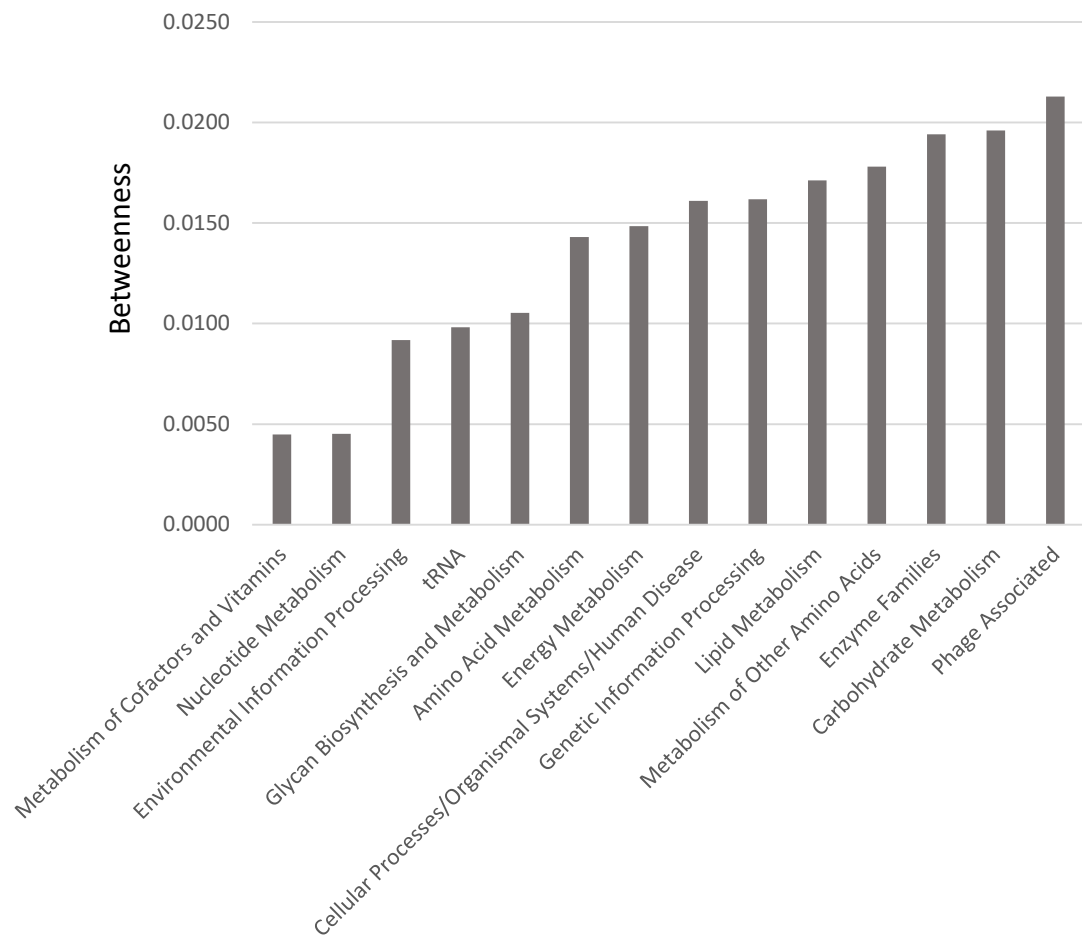**B**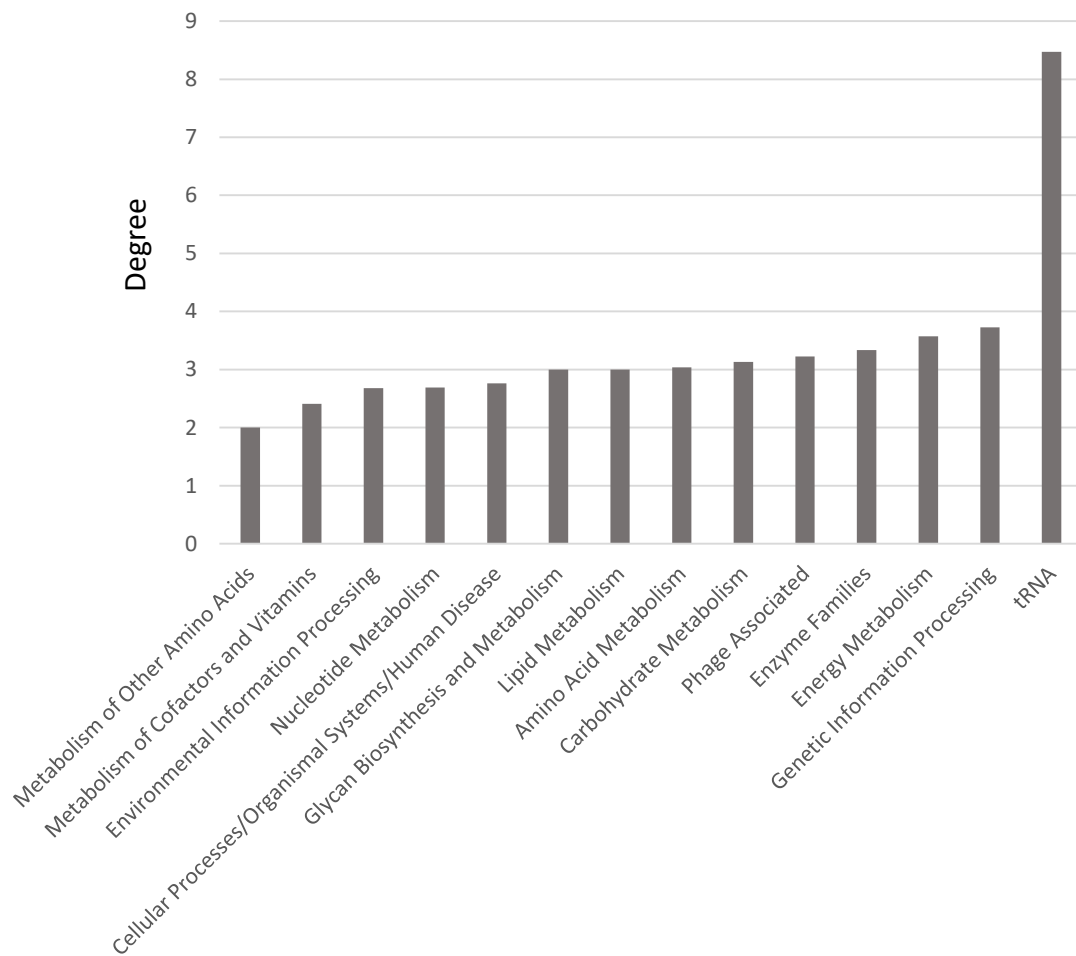

Supplement: FIG S2 [file mSystems.00729-19-sf002.pdf]

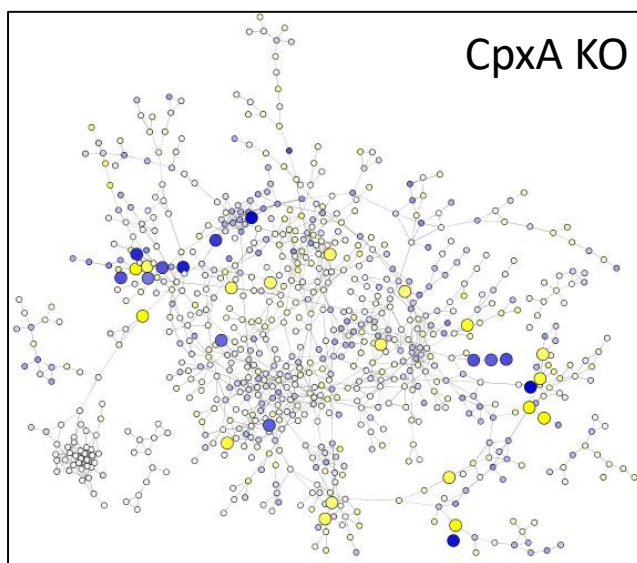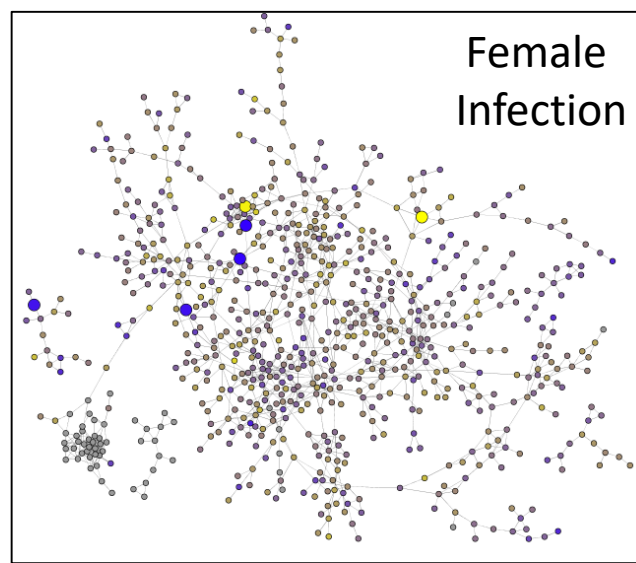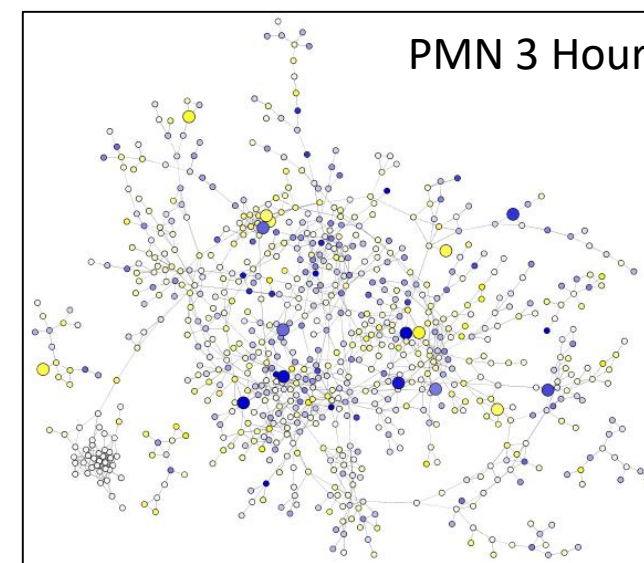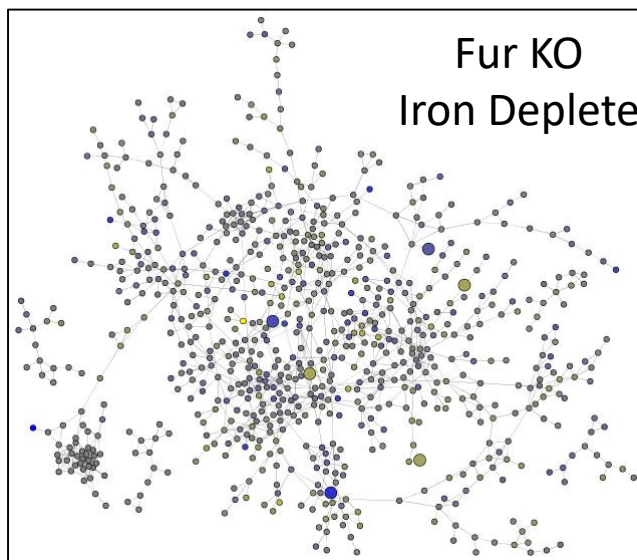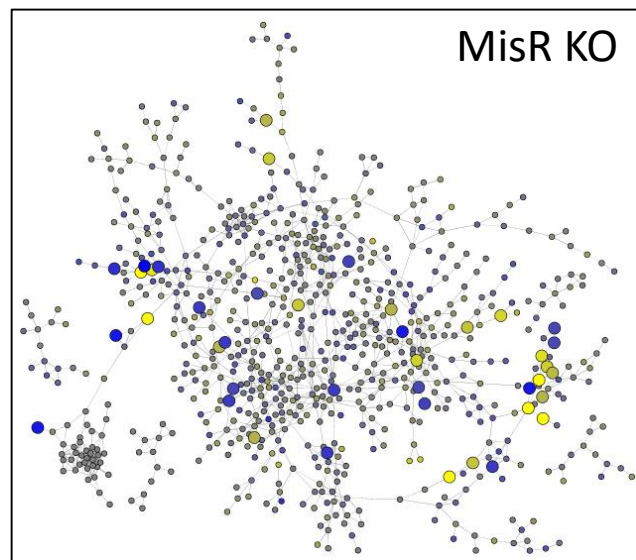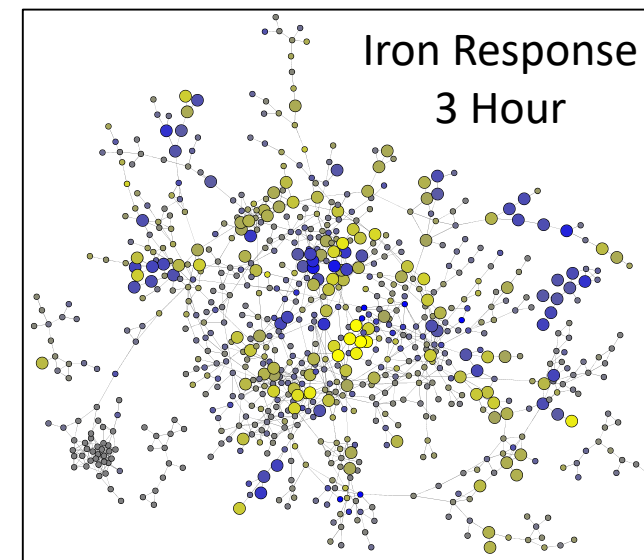

Supplement: FIG S3 [file mSystems.00729-19-sf003.pdf]

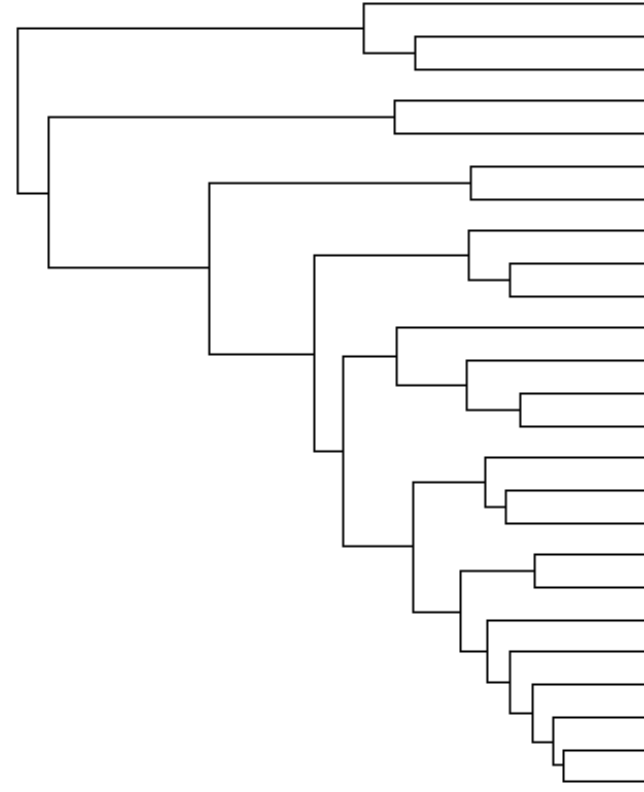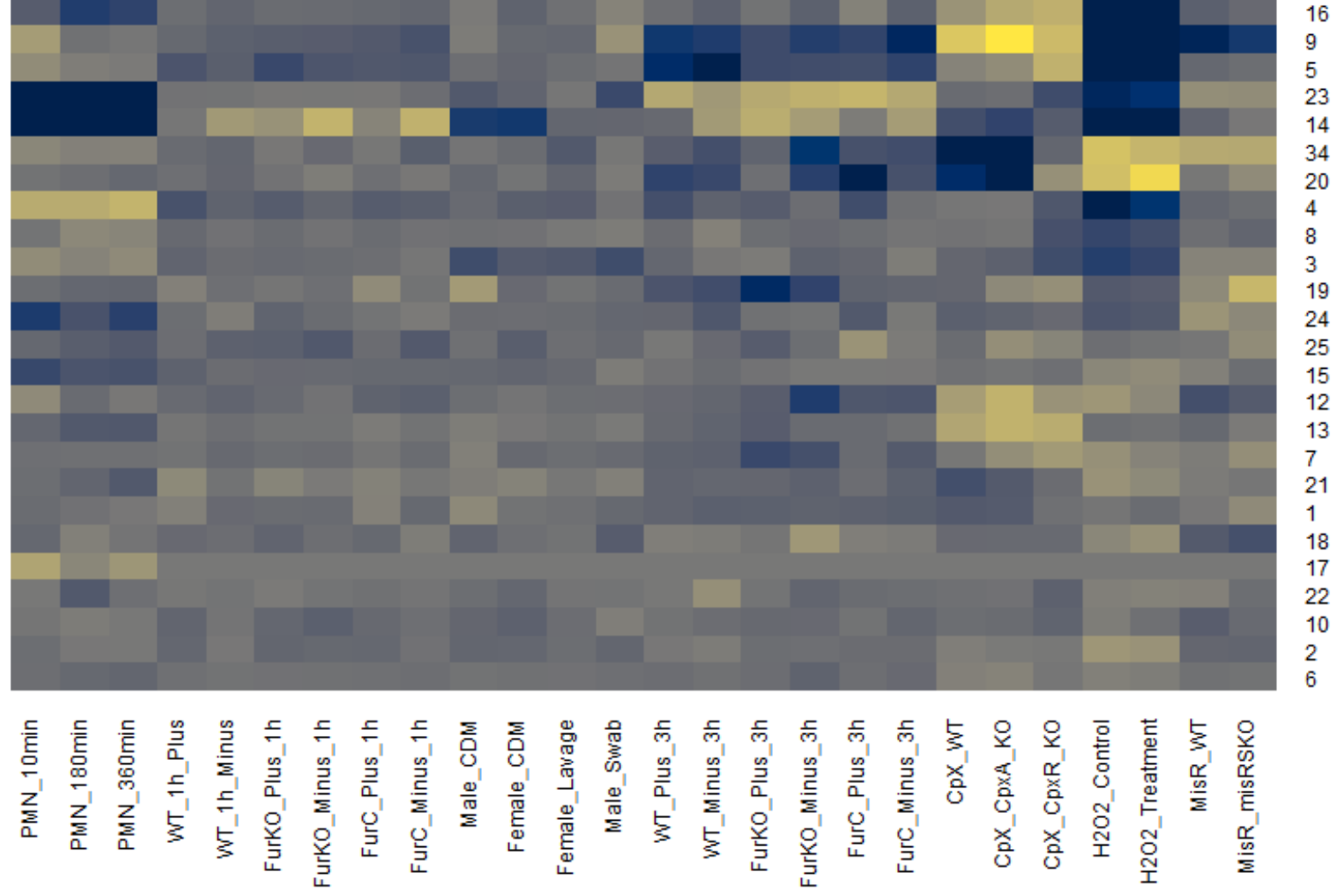

Supplement: FIG S4 [file mSystems.00729-19-sf004.pdf]
