# Supplementary material for: Global Network Analysis of Neisseria gonorrhoeae Identifies Coordination between Pathways, Processes, and Regulators Expressed during Human Infection
Source: mSystems. 2020 Feb 4;5(1):e00729-19. doi: 10.1128/mSystems.00729-19 (PMC7002116; doi:10.1128/mSystems.00729-19)
Supplement: TEXT S1 [file mSystems.00729-19-s0001.docx]

**Supplementary Methods on Computational and Network Analysis of Transcriptomic Data**

Previously existing RNA-seq files were collected from the Gene Expression Omnibus (GEO) using the accession numbers shown in Table S1. Other RNA-seq datasets were generated by our group or by our collaborators, also indicated in Table S1.

*Determining read quality*

Once all RNA-seq data was collected we used the FastQC program to determine quality of RNA reads and whether any adaptor sequences were present in the reads. FastQC is a free downloadable program with a simple graphical user interface for easy use. FastQC was downloaded from here, <https://www.bioinformatics.babraham.ac.uk/projects/fastqc/> and we examined the Per base sequence quality module to determine Phred scores for each nucleotide in the RNA reads. We also used FastQC to determine if any samples had reads with significant levels of adaptors on the end of the read. This was determined by examining the Adapter Content module. Certain samples were found to have levels of the TruSeq2-LT adaptor on their ends.

*Trimming reads*

Any nucleotide with a Phred score below 25 (corresponding to less than 99.95% confidence in the nucleotide call) was removed from the read. This was done using the Trimmomatic program. Trimmomatic is a command line program that is run using Java. Trimmomatic was downloaded from here, <http://www.usadellab.org/cms/?page=trimmomatic>, where the manual can also be found. We also used the same program to remove adaptor sequences from reads. To remove low quality nucleotides and adaptor sequences on single end files (Samples 1-55 in Table S1) the following command was run:

java –jar trimmomatic-0.36.jar SE –phred33 <input_sample_name>.fastq <output_sample_name>_Trim_LT.fastq ILLUMINACLIP:TruSeq2-LT.fa:2:30:10 LEADING:25 TRAILING:25

This command will remove nucleotides from the beginning or end of the read with a Phred score below 25. This command also removes any nucleotides that are part of the TruSeq2-LT adaptor from the reads

To remove low quality nucleotides on paired end files (Samples 56-65 in Table S1, there were no adaptors found within these reads to adaptor removal was not carried out) the following command was run:

java -jar trimmomatic-0.36.jar PE -phred33 <forward_read_input_sample_name>.fastq.gz <reverse_read_input_sample_name>.fastq.gz <output_sample_name>_TRIM_LT_paired.fastq.gz <output_sample_name>_TRIM_LT_unpaired.fastq.gz <output_sample_name>_TRIM_LT_paired.fastq.gz <output_sample_name>_TRIM_LT_unpaired.fastq.gz LEADING:25 TRAILING:25

*Alignment of reads to the N. gonorrhoeae genome*

After trimming reads were aligned to the genome of N. gonorrhoeae FA1090 (NCBI: NC_002946.2) using the Burrows-Wheeler Aligner (BWA). BWA is a command line program and was downloaded from here, <http://bio-bwa.sourceforge.net/>, where the manual can also be found. Before aligning reads, several files specific to BWA were made using the N. gonorrhoeae FA1090 fasta file (NC_002946.fna). This was done using the BWA index command:

bwa index NC_002946.fna

For single end reads the following command was run to align trimmed read files to the N. gonorrhoeae genome:

time bwa mem -t 24 NC_002946.fna <input_file>.fastq > <output_file>.sam

For paired end reads the following command was used:

time bwa mem -t 24 <reference_genome> <forward_input_file>_paired.fastq.gz <reverse_input_file>_paired.fastq.gz > <output_file>.sam

Next, aligned read files (.sam files) were used to determine gene counts. This was done using HTSeq, which uses Python. HTSeq was downloaded from there, <https://htseq.readthedocs.io/en/release_0.11.1/overview.html>, where the manual can also be found. Python can be downloaded here, <https://www.python.org/downloads/>. Alignment of .sam files also requires a .gtf file for N. gonorrhoeae which can be found here, <https://www.ncbi.nlm.nih.gov/genome/864?genome_assembly_id=300414>. To determine gene counts the following command was run:

python -m HTSeq.scripts.count -f sam -r name -s yes -a 10 -t gene -i gene_id -m union <input_file>.sam NC_002946.gtf <output_file>.txt

Raw counts for N. gonorrhoeae genes were then normalized using DESeq2, which uses R. DESeq2 was downloaded from here, <https://bioconductor.org/packages/release/bioc/html/DESeq2.html>, where the manual can also be found. The R language can be downloaded here, <https://www.r-project.org/>. The following code was used with R to normalize counts and determine gene expression levels.

library(DESeq2)

generateData <- function(){

filePath <- "<path_name_for_csv_file_containing_all_count_data>.csv"

countData <- read.table(filePath, header=TRUE, sep=",", quote="", stringsAsFactors=FALSE, comment="")

dfcountData <- data.frame(countData)

rownames(dfcountData) <- dfcountData [,1]

dfcountData [,1] <- NULL

dfcountData <- round(dfcountData)

filePath <- "<path_name_for_csv_file_condition_info>.csv"

colData <- read.table(filePath, header=TRUE, sep=",", quote="", stringsAsFactors=FALSE, comment="")

dfcolData <- data.frame(colData)

rownames(dfcolData) <- dfcolData [,1]

dfcolData [,1] <- NULL

dds <- DESeqDataSetFromMatrix(countData = dfcountData, colData = dfcolData, design = ~condition)

dds <- DESeq(dds)

dds <- estimateDispersionsGeneEst(dds)

dispersions(dds) <- mcols(dds)$dispGeneEst

notAllZero <- (rowSums(counts(dds)) > 0)

vsd <- varianceStabilizingTransformation(dds)

normalizedCounts <- assay(vsd[notAllZero,])

outputFilePath <- "<output_path_name_for_csv_file_containing_all_normalized_count_data>.csv"

write.table(normalizedCounts, file=outputFilePath, quote=FALSE, sep=",", row.names=TRUE, col.names=TRUE)

#The following line is used to compare conditions to identify differentially expressed genes, names of conditions (from the condition info file) are used in place of "A" and "B"

res <- results(dds, contrast=c("condition","A","B"))

res$names<-rownames(res)

outputFilePath <- "<output_path_name_for_csv_file_containing_differentially_expressed_genes_from_above_comparison>.csv"

write.table(res, file=outputFilePath, quote=FALSE, sep=",", row.names=FALSE, col.names=TRUE)

summary(res)

}

generateData()

*Generating and viewing networks*

Once normalized gene expression levels were determined with DESeq2 a network was made using CLR. A version of CLR comes with MINET package that can be run with R. The MINET package and manual can be found here: <https://www.bioconductor.org/packages/release/bioc/html/minet.html>. The following code was used to infer a CLR network of N. gonorrhoeae genes.

library(minet)

mutualInfo <- function()

{

#Normalized gene expression levels must be transposed, rows are conditions and genes are columns before running

filePath <- "<Transposed_gene_expression_levels>.csv"

data <- read.table(filePath, header=TRUE, sep=",", quote="", stringsAsFactors=FALSE, comment="", check.names = F)

net1 <- build.mim(data)

net2 <- clr(net1)

writePath <- "<Output_path_for_weighted_CLR_matrix>.csv"

write.table(net2, file=writePath, quote=FALSE, sep=",", col.names=TRUE, row.names=FALSE)

}

mutualInfo()

Once a weighted network was inferred a cutoff value for defining an edge in the network has to be chosen. This was done by picking several cutoff values that would lead to networks of chosen size (10,000-1,000 edges) and viewing networks to see which had good structure. To determine what cutoff values should be used to make the nine networks of 10,000 – 1,000 edges the following code was run in R.

countOnes <- function() {

inputFilePath <- "<Weighted_CLR_Matrix_file>.csv"

x <- read.table(inputFilePath, header=F, sep=",", quote="", stringsAsFactors=FALSE)

#Line below is a list of edge sizes that are desired

edges <- c(10000, 8000, 6000, 5000, 4000, 3000, 2000, 1500, 1000)

x_list <- unlist(x)

x_sort <- sort(x_list, decreasing = TRUE)

edge_1 <- x_sort[edges[1]]

edge_2 <- x_sort[edges[2]]

edge_3 <- x_sort[edges[3]]

edge_4 <- x_sort[edges[4]]

edge_5 <- x_sort[edges[5]]

edge_6 <- x_sort[edges[6]]

edge_7 <- x_sort[edges[7]]

edge_8 <- x_sort[edges[8]]

edge_9 <- x_sort[edges[9]]

included_edges <- c(edge_1, edge_2, edge_3, edge_4, edge_5, edge_6, edge_7)

outputData <- cbind(Edge_Cutoff=included_edges, edges)

print(outputData)

}

countOnes()

Next, individual files were made using these cutoff values and the full CLR matrix that can be loaded into Cytoscape for network viewing. Two file types were made, a .sif file that shows edges and a .graphml file that can be used in conjunction with other programs to determine modules in networks. To create these files the following code was run in R.

library(igraph)

filterAndCreateNetwork <- function() {

#Cutoff values determined in the Determine_edge_cutoff script should be added to the line below in place of "X"

cutoffs <- c("X")

inputFilePath <- "<Weighted_CLR_Matrix_file>.csv"

adjMatWht <- read.table(inputFilePath, header=TRUE, sep=",", quote="", stringsAsFactors=FALSE, check.names = F)

for(n in (1:length(cutoffs))) {

adjMat <- adjMatWht

minZVal <- cutoffs[n]

adjMat[adjMat < minZVal] <- 0

adjMat[adjMat >= minZVal] <- 1

modePicked <- "undirected"

g <- graph.adjacency(adjMat, mode= modePicked)

print(modePicked)

g <- delete.vertices(g, which(degree(g) == 0))

outputData <- get.edgelist(g)

outputData <- cbind(outputData[, 1], "C", outputData[, 2])

outputFilePath <- sprintf("<Output_path_for_.sif_file>_%s.sif", minZVal)

write.table(outputData, file=outputFilePath, quote=FALSE, sep=" ", row.names=FALSE, col.names=FALSE)

print(nrow(outputData))

outputFilePath <- sprintf("<Output_path_for_.graphml_file>_%s.graphml", minZVal)

write.graph(g, outputFilePath, format="graphml")

}

}

We also ran the following code in R on .graphml files to determine modules within the network.

library(ggplot2)

library(gplots)

library(igraph)

library(plyr)

findCommunities <- function()

{

#Desired module size (minimum number of genes to define a module) should be below

minModuleSize = 12

#Cutoff values determined in the Determine_edge_cutoff script should be added to the line below in place of "X"

cutoffs <- c("X")

for(i in (1:length(cutoffs))) {

cutoff <- cutoffs[i]

inputFilePath <- sprintf("<.graphml_file_from_Filter_and_Create_Networks_script>_%s.graphml", cutoff)

g <- read_graph(inputFilePath, format="graphml")

wc <- fastgreedy.community(g)

modMembership <- data.frame(GeneID=wc$names, ModuleID=wc$membership, stringsAsFactors=FALSE)

modSizes <- count(modMembership, vars="ModuleID")

mods2Keep <- modSizes[modSizes$freq >= minModuleSize, "ModuleID"]

outputData <- modMembership[modMembership$ModuleID %in% mods2Keep, ]

outputFilePath <- sprintf("<Output_path_for_module_gene_content_file>_%s_Mods.csv", cutoff)

write.table(outputData, file=outputFilePath, quote=FALSE, sep=",", row.names=FALSE, col.names=TRUE)

outputData <- modSizes[modSizes$ModuleID %in% mods2Keep, ]

colnames(outputData) <- c("ModuleID", "Size")

outputFilePath <- sprintf("<Output_path_for_module_size_content_file>_%s_Sizes.csv", cutoff)

write.table(outputData, file=outputFilePath, quote=FALSE, sep=",", row.names=FALSE, col.names=TRUE)

}

}

Once .sif files were made they were imported into Cytoscape to view the network. Cytoscape can be downloaded here, <https://cytoscape.org/download.html>, where the manual can also be found. Cytoscape makes use of a graphical user interface for ease of use.
